# Traumatic Brain Injury in a Well: A Modular Three-Dimensional Printed Tool for Inducing Traumatic Brain Injury *In vitro*

**DOI:** 10.1089/neur.2022.0072

**Published:** 2023-04-20

**Authors:** Luise Schlotterose, Megane Beldjilali-Labro, Gaya Schneider, Ofir Vardi, Kirsten Hattermann, Uzi Even, Esther Shohami, Herman D. Haustein, Yael Leichtmann-Bardoogo, Ben M. Maoz

**Affiliations:** ^1^ Institute of Anatomy, Kiel University, Kiel, Germany.; ^2^ Department of Biomedical Engineering, Tel Aviv University, Tel Aviv, Israel.; ^3^ School of Chemistry, Tel Aviv University, Tel Aviv, Israel.; ^4^ Institute for Drug Research, The Hebrew University of Jerusalem, Jerusalem, Israel.; ^5^ School of Mechanical Engineering, Faculty of Engineering, Tel Aviv University, Tel Aviv, Israel.; ^6^ Sagol School of Neuroscience, Tel Aviv University, Tel Aviv, Israel.; ^7^ The Center for Nanoscience and Nanotechnology, Tel Aviv University, Tel Aviv, Israel.

**Keywords:** 3D printing, *in vitro* model, platform development, traumatic brain injury

## Abstract

Traumatic brain injury (TBI) is a major health problem that affects millions of persons worldwide every year among all age groups, mainly young children, and elderly persons. It is the leading cause of death for children under the age of 16 and is highly correlated with a variety of neuronal disorders, such as epilepsy, and neurodegenerative disease, such as Alzheimer's disease or amyotrophic lateral sclerosis. Over the past few decades, our comprehension of the molecular pathway of TBI has improved, yet despite being a major public health issue, there is currently no U.S. Food and Drug Administration–approved treatment for TBI, and a gap remains between these advances and their application to the clinical treatment of TBI. One of the major hurdles for pushing TBI research forward is the accessibility of TBI models and tools. Most of the TBI models require costume-made, complex, and expensive equipment, which often requires special knowledge to operate. In this study, we present a modular, three-dimensional printed TBI induction device, which induces, by the pulse of a pressure shock, a TBI-like injury on any standard cell-culture tool. Moreover, we demonstrate that our device can be used on multiple systems and cell types and can induce repetitive TBIs, which is very common in clinical TBI. Further, we demonstrate that our platform can recapitulate the hallmarks of TBI, which include cell death, decrease in neuronal functionality, axonal swelling (for neurons), and increase permeability (for endothelium). In addition, in view of the continued discussion on the need, benefits, and ethics of the use of animals in scientific research, this *in vitro*, high-throughput platform will make TBI research more accessible to other labs that prefer to avoid the use of animals yet are interested in this field. We believe that this will enable us to push the field forward and facilitate/accelerate the availability of novel treatments.

## Introduction

The rapid increase in the prevalence of traumatic brain injury (TBI) with long-term neurological consequences is an alarming trend that profoundly affects society, regardless of all races and ages. It is estimated that 40 to 50 million persons are affected worldwide each year.^1^ Though clinical observations have confirmed a link between the development of neurodegenerative diseases (NDs) and TBI, the underlying mechanisms remain elusive.^2^ Although TBI poses a major problem in human health; we are limited in our ability to study it, and thus far translation to the clinic has failed, for various reasons.^3,4^

*In vivo* studies can recapitulate the different types of TBI and complexity of the whole brain.^5^ Despite their evolution and improvement (integration of advancing parameters such as age, sex, and immunity),^6,7^ animal models still suffer from interspecies differences, which may be significant in translating data into human physiology.^8^ Therefore, a gap remains in clinical trials of drugs for TBI. The use of human cells *in vitro* could provide mechanistic insight into closely mimicked biological processes.^9^ However, the *in vitro* model lacks the systemic response and complexity of the whole organism and often requires complex, unique tools to induce TBI *in vitro*.^10–13^

Therefore, in this study, we developed a modular three-dimensional (3D) printed TBI induction device (TBI-ID) that can fit to any standard culture tool (such as: 96- to 6-well plates, multi-electrode array [MEA], Transwell^®^, etc.), and given that it is made by 3D printing, it can be easily made anywhere without special knowledge. Moreover, we demonstrate the abilities of the device to induce TBI on different cell types, including neuron-like cells (SH-SY5Y cells) and the endothelium, and assess how repetitive, rapid pressure change affects cell morphology, cellular functionality, and apoptosis. A TBI protocol of repetitive injuries can also be applied in the present device. This is a major problem in different combat-related TBIs as well as in several contact sports (e.g., boxing, football, etc.).^14^ Overall, we present a modular TBI-ID that can be used with a variety of lab tools, including physio- and/or pharmacological manipulations, to mimic the environmental effect and affect outcome after the induced injury. This is a high-throughput system that can promote the study of TBI by making it more accessible to other labs.

## Methods

### TBI device

The TBI device was a 12-cm-high hollow tube that was 3D printed out of long-term biocompatible resin, Dental LT Clear with the Form2 3D-printer (Formlabs, Somerville, MA). The head of the tube was detachable and changeable according to the plate size used (6-, 12-, 24-, or 96-well). A commercially available double rubber band No. 14 (50.0 × 2.0 mm) was attached on the top of the tube, using the printed holders and secured with a second rubber band, as shown in Supplementary Figure S2. The weight used was a 36.74-g metal cylinder with a length of 6 cm and a diameter of 1 cm. The device was used for 1 × hit or 3 × hit (consecutively), and all time points were measured starting from the last impact.

### Human neuroblastoma SH-SY5Y cell cultures

Human neuroblastoma SH-SY5Y cells, infected with α-syn(A53T)-GFP adeno-associated virus, were generated by Yifat Weiss, from Ashery's lab. Cells were grown in a culture medium (1:1 RPMI/F12 [01.100-1A, 01-095-1A]; Sartorius, Beit Haemek, Israel), 10% fetal bovine serum (FBS; Sartorius), 0.15% sodium bicarbonate (BI; 7.5%, 03-040-1B), 1% GlutaMAX (Gibco 35050038; ThermoFisherScientific, Waltham, MA), 100 U/mL of penicillin, and 100 μg/mL of streptomycin (BI, 03-031-1B) and incubated at 37°C/5% CO_2_. For TBI experiments, cells were differentiated to neuron-shaped-like as detailed in the section on cell differentiation below.

### SH-SY5Y cell differentiation

SH-SY5Y cells were differentiated over 11 days. Twenty-four-well plates were coated with 10 mg/mL of poly-D-lysine (PDL; ThermoFisherScientific) and subsequently with 4 μg/mL of laminin (Sigma-Aldrich, St. Louis, MO). Cells were then seeded on coated plates (10,000 cells per well) and allowed to grow for 24 h. Next, 50% of the medium was changed to culture medium containing 10 μM of retinoic acid (Sigma-Aldrich). Medium was refreshed after 48 h. On day 8 of the differentiation, all the medium was changed to FBS-free culture medium containing 2 ng/mL of brain-derived neurotrophic factor (PeproTech, Inc., Cranbury, NJ). Differentiation was completed after 3 more days of incubation.

### Rat primary cortical cell culture

Dissociated neuronal cultures were obtained from cerebral cortices of newborn Sprague-Dawley rats at days P3–P4 as previously reported.^15^ All experiments were approved by the local veterinary authority and the animal ethics committee of Tel Aviv University (approval ethic no.: 01-19-079) and performed in accordance with Israeli law. All efforts were made to minimize animal suffering and reduce the number of animals used. All chemicals were purchased by Sigma-Aldrich unless stated otherwise. Enzymatically dissociated cells (10 mg of trypsine 600 U, 1.5 mg of DNAse, and 2.5 mg of trypsine inhibitor) were plated at a density of 400,000 cells per well (values sampled form *n* ≥ 3 cultures) on 20 μg/mL of laminin-coated six-well plate MEAs. For the first 24 h, neurons were left in plating media, composed of Neurobasal-A medium (Gibco 10888022; ThermoFisherScientific), 5% FBS (BI, 04-007-1A), 0.5% penicillin-streptomicin (BI, 03-031-1B), 1% GlutaMAX (Gibco 35050038; ThermoFisherScientific), and 2% B27 (Gibco 17504044; ThermoFisherScientific).

Culture medium (same composition without FBS) was renewed after 2 days from seeding and contained additionally an inhibitor of glial cell proliferation, 10 μM of fluorodeoxyuridine (F0503-100MG), and gentamycin (Gibco 15710049, 10 mg/mL; ThermoFisherScientific). Cultures were used 12 days after seeding.

### Endothelial cell culture

Human umbilical vein endothelial cells (HUVECs; PromoCell GmbH, Heidelberg, Germany) were used to test the impact on cell-barrier properties. HUVECs were cultured in low-serum endothelial cell growth medium (PromoCell) at 37°C with 5% CO_2_ in a humidifying incubator and used at passage p4–p6. Cells were cultured on transparent polyethylene terephthalate Transwell supports (0.4-μm pore size; Greiner Bio-One, Kremsmünster, Austria), coated with Entactin-Collagen IV-Laminin (E-C-L) Cell Attachment Matrix (Merck Millipore, Burlington, MA) diluted in Dulbecco's modified Eagle's medium (10 μg/cm^2^) for 1 h before seeding, at a density of 40,000 cells/cm^2^, and grown for 3 days.

### Cell viability assay

Cell viability was analyzed by determining the amount of dead and live cells. Propidium iodide (PI; Sigma-Aldrich P4170) was used to stain dead cells and Calcein acetoxymethyl ester (Calcein-AM; Invitrogen, ThermoFisherScientific, Carlsbad, CA) for living cells. Twenty-four hours after TBI was induced, treated and control cell cultures were incubated with 2.5 μg/mL of Calcein-AM, 40 μg/mL of PI, and 5 μg/mL of Hoechst (ThermoFisherScientific) simultaneously for 30 min at 37°C. Cells were then visualized with an Olympus inverted fluorescence microscope equipped with multi-variant fluorescence filters (Olympus Corporation, Tokyo, Japan). Cell proliferation was monitored using AlamarBlue Cell Viability Reagent (Invitrogen, ThermoFisherScientific DAL1025), according to the manufacturer's instructions. Fluorescent intensity was measured with the Tecan Spark multi-mode microplate reader and normalized according to the blank and control wells (Tecan, Männedorf, Switzerland). Cell adherent was analyzed using ImageJ,^16^ quantifying the uncovered area of treated wells compared to controls.

### High-speed imaging

For analysis of the impact process, high-speed video was used based on a monochrome Photron AX200 camera matched to a Navitar X12 long-working-distance microscope. Imaging was conducted at 10 kfps under backlighting by a focused 30-W white LED light source. Because of the higher frame rate, the total image was around 18.0 × 15.4 mm (896 × 768 pixels) at a resolution of 20 microns per pixel. For typical imaging, 10-ms exposure time was sufficient to prevent motion blur, though the addition of particles to the liquid (mean diameter: 11 ± 8 microns, not shown) required exposure times down to 333 ns. Particles were observed to track the surface of the bubble well and show the velocity decay (∼1/r^2^) farther away.

### Multi-electrode array recordings and data analysis

Commercial six-well-plate MEAs (Multichannel Systems; Reutlingen, Germany), each containing an array of nine embedded gold electrodes (30 μm in diameter, 200 μm apart), were used to non-invasively monitor the electrical activity of neuronal networks, at different time points, post-TBI “1 × hit” or “3 × hit.” The day before cell seeding, MEAs were coated with PDL 50 μg/mL (Gibco A3890401, 0.1 mg/mL) overnight at 4°C, followed up by laminin 20 μg/mL (Sigma-Aldrich L2020, 1.6 mg/mL) for 2 h at 37°C in a cell-culture incubator. An amount of 4 × 10^5^ primary cortical neurons were plated in each well. Cells were grown in a 37°C incubator (5% CO_2_ in air), and 50% of the medium was refreshed every 2 days. At day 12, spontaneous neuronal activity (20-kHz sampling rate; 5-min recording), including firing- and burst-rate activity, were recorded upon mounting the MEA plate into the recording system. Detected spikes were sorted and analyzed using a MATLAB program (The MathWorks, Inc., Natick, MA).

Raw data were filtered with a bandpass filter between 300 and 1100 Hz, and the threshold for detecting spikes was determined to be 4.5 times the standard deviation (SD) of the average of the entire signal. Only active electrodes, defined as ≥15 spikes/min (0.25 Hz), were used for analysis. Bursts were defined as three or more spikes, with a ≤300-ms interspike interval on the same burst.

### Transepithelial endothelial electrical resistance measurement

Barrier properties of the endothelial monolayer were evaluated by transepithelial endothelial electrical resistance (TEER) measurements, before and 1 and 24 h after TBI. TEER was measured with the Millicell ERS-2 Voltohmmeter (Merck Millipore). TEER values (Ω cm^2^) were calculated and compared to those obtained in a Transwell insert without cells, considered as a blank, in three different individual experiments, with two inserts used for each condition (control; 1 × hit and 3 × hit).

### Imaging (optical + epifluorescence)

Light microscopy images were captured by a Nikon Eclipse TS100 microscope (Nikon, Tokyo, Japan). Fluorescence images were captured using the Olympus IX83 inverted microscope and Olympus FV3000 confocal laser scanning microscope and were imaged with a 10 × or 20 × objective.

### Statistical analysis

All experiments are performed in at least three independent replicates. The results shown are presented as mean ± SD from individual experiments. Data from different cell lines and conditions were compared using appropriate statistical tests. Using GraphPad Prism software (version 9.4.1; GraphPad Software Inc., La Jolla, CA), *p* values were calculated for differences between multiple groups by one- or two-way analysis of variance, followed by Tukey's multiple-comparison test. A statistically significant difference between two data sets was assessed, and *p* < 0.05 was considered statistically significant.

## Results

### Traumatic brain injury induction device establishment

**FIG. 1.**
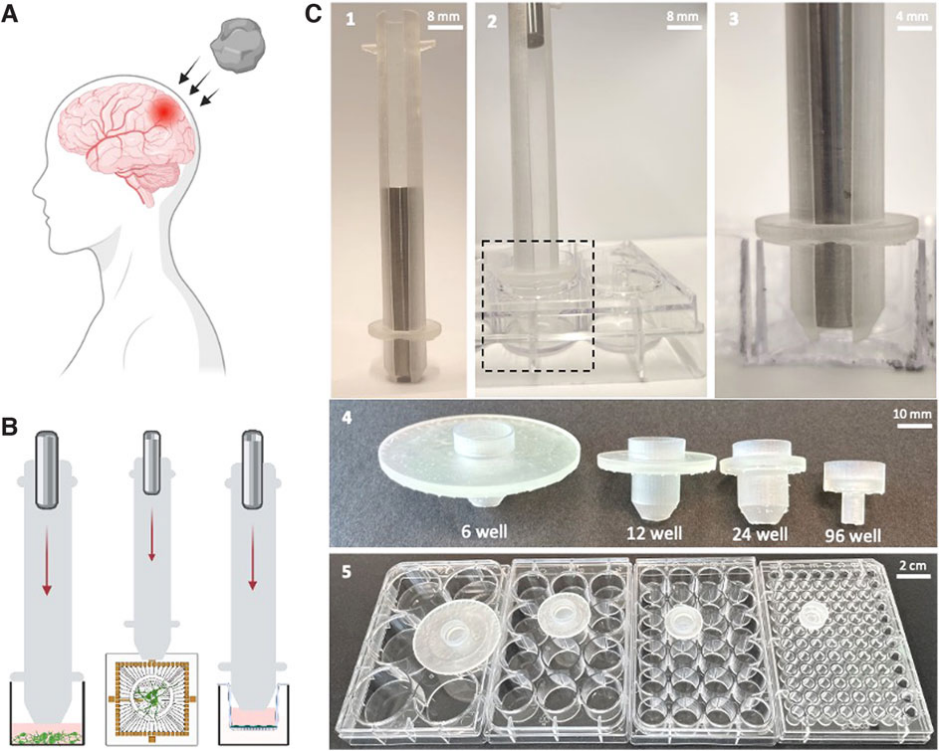
TBI-device, modularity. (**A**) Illustration of injury during TBI. (**B**) Examples of different usable systems (standard well-plate, MEA plate, or inserts). (**C**) TBI-ID and its changeable heads. (C1-3) Vertical cross-section of TBI-ID tube to visualize the movement of the weight in the tube, (C2) positioned in a well plate. (C3) Zoom of the end position of the weight inside the tube reaching the narrow tip of the head. (C4) Different-sized heads (for 6-, 12-, 24-, and 96-well plates) and (C5) their fitting in a standard well plate. MEA, multi-electrode array; TBI, traumatic brain injury.

The goal of this study was to develop a modular, simple, and easy-to-use platform to induce TBI *in vitro,* which can be used in compliance with any standard culture tool and would allow for studying its effect on the cellular and molecular response to injury under various experimental conditions. To do so, we designed and built a TBI-ID that translates the kinetic energy generated by a falling weight to pressure shock and which induces injury to cells (Fig. 1A,B,C1-3). To provide compatibility to standard lab culture tools, we designed a changeable head that can fit into 96-, 24-, 12- and 6-well plates, MEAs, and Transwell (Fig. 1C4-5 and Supplementary Figs. S1 and S2). The “main tube” is slightly smaller than its corresponding frame on the head. Therefore, the different heads can be changed simply by fitting it to the main tube without the need for further adjustments.

**FIG. 2.**
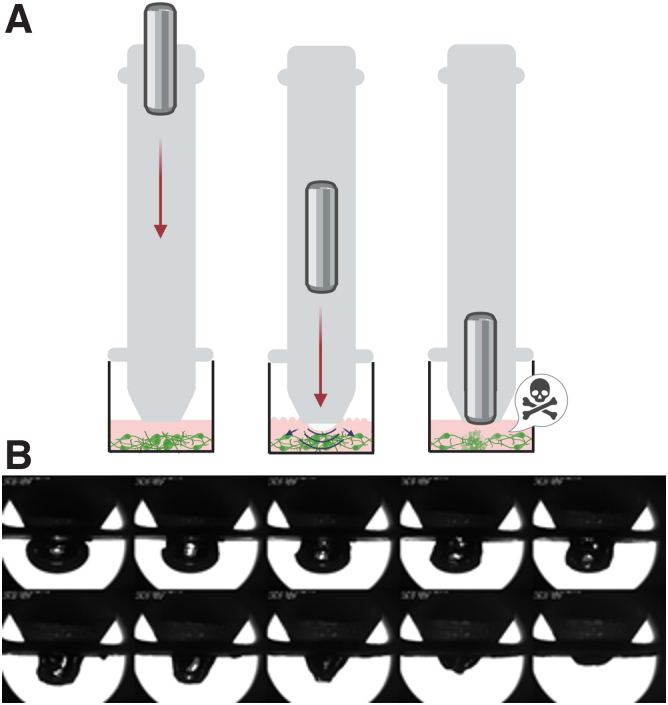
Mechanism of impact, (**A**) Projection of the weight drop and simultaneous formation of an air bubble leading to cell injury. (**B**) High-speed images to evaluate the properties of air-bubble formation and collapse of the lower bubble front (1.2 ms from frame to frame).

**FIG. 3.**
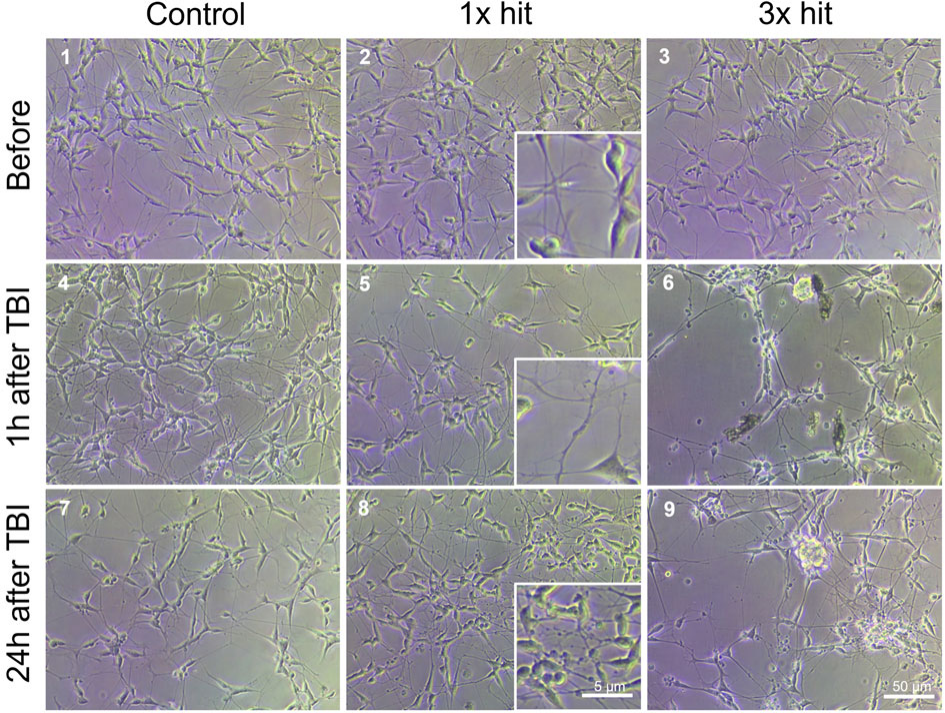
Morphology of differentiated SH-SY5Y cells changes after impact. Representative images by light microscopy of differentiated SH-SY5Y cells before TBI (1,2,3), 1 h (4,5,6), and 24 h (7,8,9) after impact, (1,4,7) control (no injury), (2,3,8) 1 × hit, and (3,6,9) 3 × hit. Changes in cell morphology are enlarged. *N* = 3 individual experiments. Scale bar = 50 and 5 μm, respectively. TBI, traumatic brain injury.

**FIG. 4.**
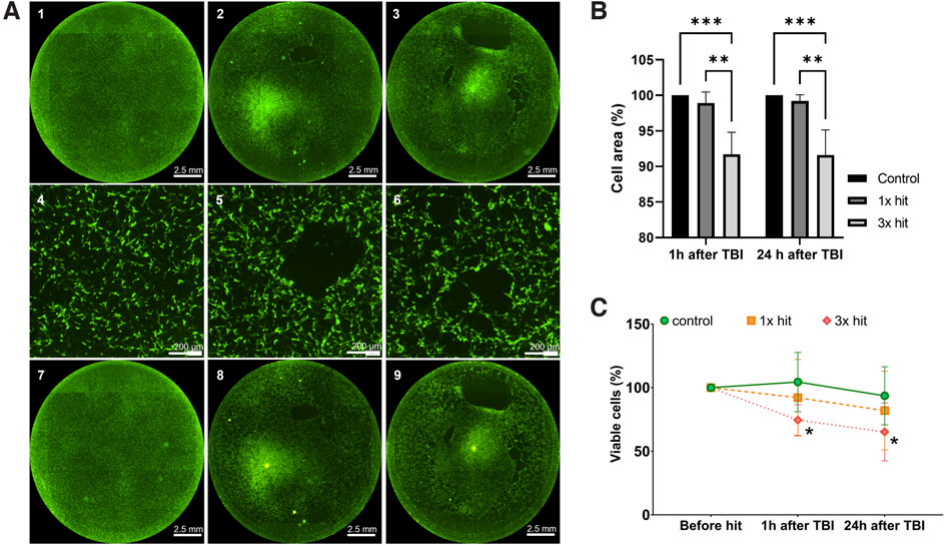
Viability of differentiated SH-SY5Y cells, (**A)** Representative fluorescence microscopy images of differentiated SH-SY5Y cells, 1 h (1–6) and 24 h (7–9) after impact, (1,4,7) control (no injury), (2,5,8) 1 × hit, and (3,6,9) 3 × hit. (4–6) Higher magnification of impact areas. Scale bar, 2.5 cm and 200 μm. (**B**) Quantification of cell-free area presented in percentage (control = 100%). (**C**) Cell viability plotted in percentage of quantity before treatment 1 h and after 24 h post-TBI. *N* = 3 individual experiments. Data are represented as mean ± SD: *p < 0.05, **p < 0.01, ***p < 0.001. SD, standard deviation; TBI, traumatic brain injury.

**FIG. 5.**
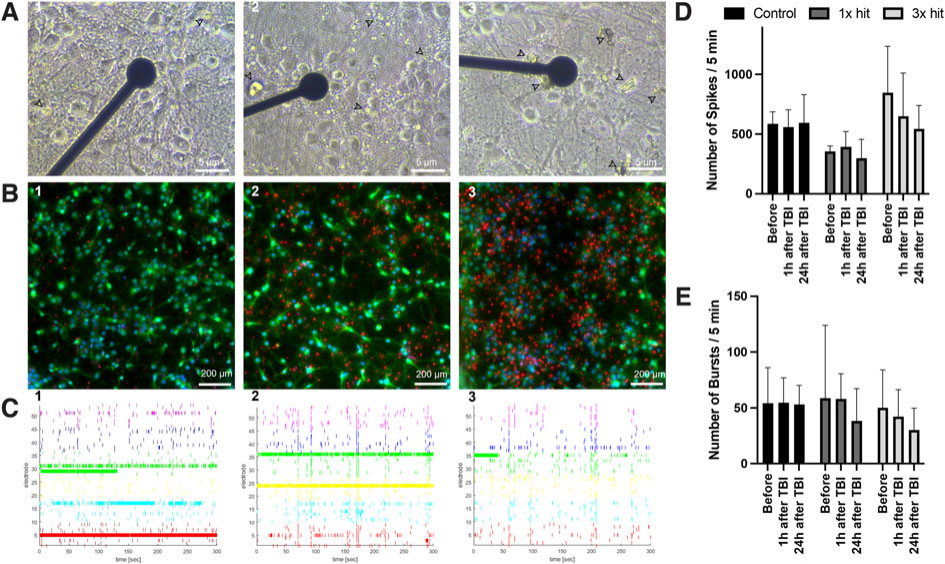
Spontaneous activity of cortical neurons recorded with MEA. (**A**) Visualization by light microscopy of primary cortical neurons (DIV14) over an MEA 24 h after injury: (1) control (no-injury); (2) injured neurons after 1 × hit; and (3) neurons after 3 × hit. Electrodes are 30 μm wide; electrode spacing is 200 μm. Scale bar = 5 μm. (**B**) Live/dead assay 24 h after injury: (1) control (no-injury); (2) injured neurons after 1 × hit; and (3) neurons after 3 × hit, live (Calcein-AM-positive green fluorescence) and dead (PI-positive red fluorescence). Scale bar = 200 μm. (**C**) Representative raster plot of spikes from >20 electrodes at 14 DIV, showing the pattern and density of spikes during 5-min recording. In all graphs, the voltage-threshold-based algorithm runs over 20,000-Hz high-pass–filtered traces and is adjusted to 4.5 times the SD of the noise. (**D**) Number of spikes per active electrode. Active electrodes were defined by presenting ≥15 spikes/min; (**E**) Number of bursts; Bursts were defined as three or more spikes with a 300-ms interspike interval. *N* = 3 individual experiments. The same batch of neurons was recorded for all time points, and six wells were sampled for each line at each time point. Data are represented as mean ± SD. Calcein-AM, Calcein acetoxymethyl ester; MEA, multi-electrode array; PI, propidium iodide; SD, standard deviation; TBI, traumatic brain injury.

Once the TBI-ID was assembled with the desired head, it was placed in a well with its tip just touching the surface of the medium (Fig. 1C-5). To induce TBI, a weight was allowed to free-fall from at the top of the tube until reaching a narrowing at the tip of the head, to ensure that the weight would not touch the cells (Fig. 1B,C2–3). The intensity of the hit could be tuned by adjusting the weight fall from different heights, or to increase the kinetic energy by adding a rubber band (Supplementary Fig. S2). Moreover, the energy of the fall (which would hit the liquid) could be calculated and is presented in Supplementary Table S1.

As shown in Figure 2, the weight drop induces TBI by creating hydrostatic pressure, which is transmitted to cells. To characterize the dynamics of the TBI induction, we used a superspeed camera (down to a 333-ns exposure time), which enabled us to identify how the drop would create an air bubble, which collapsed once the weight impacted the tube (Fig. 2B). By analyzing the video from the high-speed camera, we calculated that the growth speed of the air bubble was typically around 40 mm/s, whereas its collapse was 1–2 orders of magnitude faster. Thus, the steady pressure shock, direct mechanical hit, and its associated shock waves are found to be minor compared to the sudden negative pressure impacting cells on the surface because of the bubble collapse at the nozzle. In other words, the main pressure change causing damage to the neurons on the surface is a rapid pressure drop during the bubble collapse, similar to the well-known cavitation phenomenon.^17^

The reader is referred to a review of the effects of this phenomenon on tissue and specifically in TBI.^18,19^ It is important to note that as the bubble collapses quite symmetrically, and not adjacent to the bottom surface, it does not cause damage by the typical re-entrant jet impingement (i.e., direct liquid impact). Rather, it is the pressure transition wave, going rapidly from low positive to high negative values, that is known to cause damage. Moreover, although the present pressure values were lower than those in the above-mentioned literature, they were applied directly to the exposed tissue and were shown to be sufficient to cause damage. As shown in Figure 2B, an average bubble front velocity of ∼1 m/s generated negative pressures of 10 Pa on the bottom surface. However, dynamic image analysis of several other experiments revealed much higher instantaneous pressures, as high as 1000 Pa. These high values occur when bubble collapse synchronizes with the weight rebound (rise), causing the bubble to collapse as it is being sucked into the nozzle exit where instantaneous velocities of 10 m/s cause high pressures on the surface (Fig. 2B).

### Traumatic brain injury induction device validation

In order to validate the TBI-ID, and ensure that it could injure cells, we cultured neuronal-like cells (Fig. 3, Supplementary Fig. S3; differentiated human neuroblastoma SH-SY5Y cells) in a 24-well plate and monitored morphological changes attributable to the hits generated by the TBI-ID. As shown in the brightfield images (Fig. 3, insets 5 and 8**)**, 1 × hit of TBI-ID generated morphological changes as soon as 1 h after the hit, compared to control samples. These morphological changes can be identified as axonal swelling, which is an indicator for brain injury, associated with axon dysfunction and, in multiple NDs, an early pathological finding.^20^

The TBI-ID is not limited to one-time use, and can be used multiple times, to mimic multiple brain hits, as often occurs in human TBI. To create multiple hits, the weight was allowed to free-fall three times on the sample. Similarly, to 1 × hit, we could observe axonal swelling, but in contrary to 1 × hit, clusters of dead cells were identified (Fig. 3, insets 6 and 9). In addition, after 24 h, cells that were hit once continued to develop axonal swelling, whereas those hit three times became more aggregated and many died.

In addition to brightfield images, we used fluorescence microscopy to quantify demerged area and cell viability. As shown in Figure 4 (insets 2 and 3), both the 1 × hit and 3 × hit caused a reduction in coverage area, where for the 3 × hit, reduction was much more significant; however, no significant changes in coverage area were observed over time (Fig. 4B). In addition to percent coverage area, we quantified the number of living cells 1 and 24 h after impact (Fig. 4C). The 1 × hit did not significantly reduce the percentage of living cells, whereas the 3 × hit resulted in a significant decrease in viable cells (Fig. 4C). These results demonstrate that, by tuning the amount of hits, we can control the severity of the injury and amount of cell death.

### Functional assessment as a results of traumatic brain injury induction device

Modularity of the platform enables one to monitor how the injury affects neuronal functionality, by using MEA and monitoring changes in neuronal electrical activity (Fig. 5). Primary neurons cultured and injured on top of the MEA showed similar morphological changes (Figs. 2 and 5A) and viability (Fig. 5B) to those (SH-SY5Y) cultured on standard 24-well plates (Figs. 3 and 4). Figure 5C shows a typical electrical activity recorded from six-well plates (nine electrodes per well). As presented in the raster plots, neurons showed an extensive electrical activity that changed significantly over time after the injury. The number of spikes per active electrode showed a decrease over time in the injury condition of 1 × hit and 3 × hit of 16% and 35.8%, respectively, whereas the control condition showed consistency with a slight increase of 1.43% in the number of spikes after 24 h (Fig. 5C,D).

Given that neurons in the network tended to fire in bursts, we analyzed and compared the burst-firing activity between our injured and control neurons (Fig. 5E). Average rate of burst discharges was higher in control compared to 1 × hit and 3 × hit (53.0 vs. 38.4 bursts/5 min and 30 bursts/5 min), respectively, at 24 h post-impact. Depending on the number of hits performed on the neurons, two patterns of firing behavior were observable over time. The first effects of trauma were noticeable only 24 h after receiving 1 × hit. Neurons suffering repetitive hits, in contrast, displayed a different firing behavior as early as 1 h post-trauma (Fig. 5D,E).

### Platform modularity

**FIG. 6.**
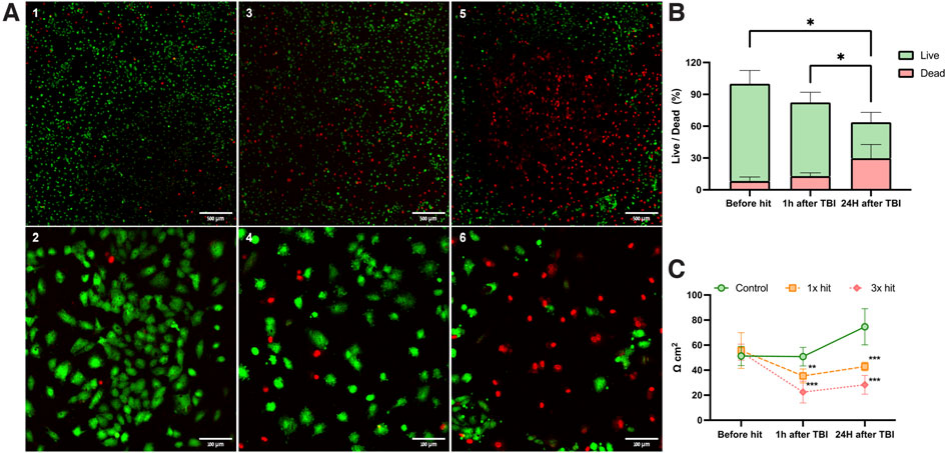
Endothelial barrier grown on the insert after TBI. (**A**) Live/dead cell assay by Calcein-AM/PI double staining of HUVEC cells (P5) cultured in Transwell at 24 h after injury. (1,2) Control (no-injury), (3,4) injured neurons after 1 × hit, and (5,6) injured neurons after 3 × hit. Green (Calcein-AM) and red (PI). Scale bar (1,3,5), 500 μm and (2,4,6) 100 μm. (**B**) Results of live/dead assays showing the percentage of viable HUVECs in Transwell 24 h after injury. Control (no-injury) shows baseline live (Calcein-AM-positive green fluorescence) and dead (PI-positive red fluorescence). Injured HUVECs after 1 × hit showed an increase in the dead cell population. Injured neurons after 3 × hit showed significant cell death, as compared to control and 1 × hit. (**C**) Plot showing TEER values of HUVECs cultured before and after TBI (1 and 24 h) on Transwell. *N* = 3 individual experiments in technical replicates. Data are represented as mean ± SD; *p < 0.05, **p < 0.01, ***p < 0.001. Calcein-AM, Calcein acetoxymethyl ester; HUVECs, human umbilical vein endothelial cells; PI, propidium iodide; SD, standard deviation; TBI, traumatic brain injury; TEER, transepithelial endothelial electrical resistance.

Given that TBI affects multiple cell types, we decided to test our modularity TBI-ID on commercially available platforms, which allow their functionality assessment. To this end, HUVEC cells were cultured on Transwell (Fig. 2B), injured with the TBI-ID, and their permeability was assessed 24 h later using TEER measurements. Calcein/PI staining (Fig. 6A) showed that 24 h after injury, there were increased apoptotic events relative to the control (Fig. 6A, insets 3 and 5). Whereas no significant difference between the percentage of viable HUVECs in control and 1 × hit samples were noted, a significantly increased apoptosis was observed (Fig. 6B) in the 3 × hit group as compared to control and to the culture subjected to the 1 × hit (21.5% and 16.9% respectively).

In addition, TEER measurements were used to assess the barrier function of HUVEC cells over the course of the observation period, at 1 and 24 h post-injury. Significant differences were found between TEER values 1 h after trauma (50.73 ± 7.40 Ωcm^2^ control, 35.35 ± 5.62 Ωcm^2^ for 1 × hit, to 22.45 ± 8.64 Ωcm^2^ for 3 × hits). These differences were accentuated 24 h after the trauma (74.64 ± 14.45 Ωcm^2^ control, 42.85 ± 3.48 Ωcm^2^ 1 × hit, and 28.31 ± 7.51 Ωcm^2^ 3 × hit; Fig. 6C).

## Discussion

Despite more than three decades of translation of pre-clinical research to human TBI, all phase 3 clinical trials were disappointing. The question of why promising therapeutics that aim for the early treatment of severe TBI fail in translation to clinical practice is still being asked. Basic research continues to document the complexity of post-TBI temporal changes in the brain, which involve neuronal networks, neuroinflammatory responses, and involvement of the neurovascular unit.

We have recently developed a modular, easy-to-fabricate, 3D-printed device that produces hydrostatic pressure over cells in culture, resulting in mechanical perturbation of cells. The rapid pressure changes initiate cellular events typical to the pathophysiology of TBI, thus making this device an ideal *in vitro* model and a novel research tool for he study of intrinsic cellular responses of the brain to the mechanical injury imposed by an external force. We have demonstrated, in our model, that trauma induction fits any standard culture tools, is applicable to any isolated cell types, and can be further used in mixed cultures. The device was proven to have low “user-to-user variability” by incorporating a shock sling part to maintain the rubber armed. Moreover, multiple users repeated the same experiment and low SDs were obtained, strengthening the robustness and consistency of the model. Further, it is a high-throughput platform that allows for the studying of basic cellular mechanisms of injury on one hand and serves as a tool for evaluating potential therapeutic modalities, on the other.

The device is composed of three main parts—the tube, a rubber, and a modular “head”—with an additional shock sling tool (Supplementary Figs. S1 and S2). TBI in humans is categorized as mild, moderate, or severe based on its severity, whereas device-induced injury severity can be adjusted and monitored by controlling the impact energy. A wide range of pressures, from a few pascals to several kPa, are used for the modeling of forces, causing an injury *in vivo* and *in vitro*^21–23^ (Supplementary Table S2). Here, high-speed imaging (Fig. 2) demonstrated a pressure of 1 kPA, with a cavitation process of the collapsing of the local bubble at a velocity of 10 m/s. By adjusting the metal weight choice, the height it is dropped from, or the acceleration of the weight (using a rubber band), it is possible to achieve a broad spectrum of pressure.

Additionally, this model can replicate another important clinical parameter—the number of hits delivered to the culture, which simulates repetitive TBI.

The benefit of using 3D printing is the ability to deliver all the parts already designed/suit made, while at the same time allowing, if necessary, their modification and optimization to fit more specific and unique culture systems. Here, we demonstrated that TBI-ID on neuronal-like cells caused axonal swelling, which is among the most common and prominent pathological features of TBI. It is also observed in all severities of TBI and may represent a key pathological in mild TBI.^24^ In addition, multiple hits present severe injuries. In fact, a depletion of cell surface area was observed, which could be attributablee to an alteration in cell adhesion molecules^25,26^ or membrane disruption.^27^ Those phenomena occur in various models of TBI, both *in vitro* and *in situ*, upon physical impact.^28^ Nevertheless, little is known about the pathway leading to pathological progression and their potential roles in post-injury recovery. In addition, axonal swelling and synaptic alterations can lead to changes in neuronal connectivity and are likely to contribute to the effects of TBI on cognitive function.^25^

This was shown on three different cell types (endothelium [HUVEC] and primary neurons; neuronal-like cells [SH-SY5Y]), which are the primary targets for TBI studies. Morphological and functional changes exhibited by cells after TBI-ID correlated with those observed by other studies, using other *in vitro* TBI induction platforms. We identified an increase in apoptosis,^29^ appearance of axonal swelling,^30–32^ decrease in neuronal activity,^33^ and increase in endothelial permeability.^34,35^ Moreover, we showed that repetitive impacts significantly increased cellular susceptibility to injury, both in the phenotype and time scales of the cellular response. The latter observation is of specific interest for human TBI given that multiple concussions appear to be a risk factor for cognitive impairment and mental health problems in persons such as athletes and military personnel.^36–38^ The *in vitro* TBI model presented here is derived from a pulse of hydrostatic pressure that differs from human TBI induced by a direct mechanical hit or shock waves. Yet, as reported here, it leads to similar cellular and molecular responses.

Overall, we present a modular, easy-to-produce, and easy-to-use TBI-ID that can induce TBI on any standard (and non-standard) tissue-culture platforms. We believe that our throughput platform will expedite our understanding of TBI pathophysiology and provide better tools for developing and evaluating novel interventions to improve recovery after TBI.

## Supplementary Material

Supplemental data

Supplemental data

Supplemental data

Supplemental data

Supplemental data

## Data Availability

The data that support the findings of this study are available from the corresponding author upon reasonable request.

## Abbreviations Used

3D: three-dimensional
BI: 0.15% *s*odium bicarbonate
Calcein-AM: Calcein acetoxymethyl ester
FBS: fetal bovine serum
HUVECs: human umbilical vein endothelial cells
MEA: multi-electrode array
ND: neurodegenerative disease
PDL: poly-D-lysine
PI: propidium iodide
SD: standard deviation
TBI: traumatic brain injury
TBI-ID: TBI induction device
TEER: transepithelial endothelial electrical resistance
