# Supplementary material for: Traumatic Brain Injury in a Well: A Modular Three-Dimensional Printed Tool for Inducing Traumatic Brain Injury In vitro
Source: Neurotrauma Rep. 2023 Apr 20;4(1):255–66. doi: 10.1089/neur.2022.0072 (PMC10122253; doi:10.1089/neur.2022.0072)
Supplement: Supplemental data [file Suppl_FigS2.pdf]

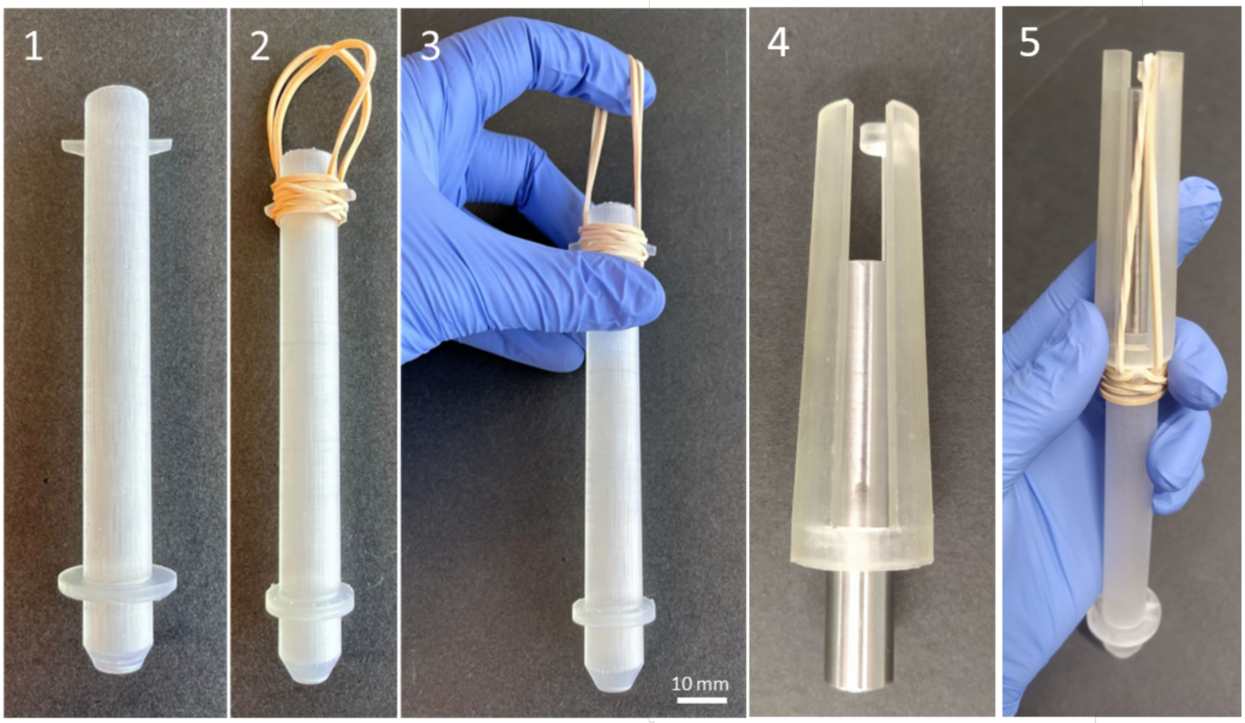


**SI Fig. 2.** Full view of the TBI-device (1) without elastic, (2) with elastic, (3) with stretched elastic, (4) sling choc device and the full TBI-device with sling choc and elastic (5).
