# Supplementary material for: Traumatic Brain Injury in a Well: A Modular Three-Dimensional Printed Tool for Inducing Traumatic Brain Injury In vitro
Source: Neurotrauma Rep. 2023 Apr 20;4(1):255–66. doi: 10.1089/neur.2022.0072 (PMC10122253; doi:10.1089/neur.2022.0072)
Supplement: Supplemental data [file Suppl_FigS1.pdf]

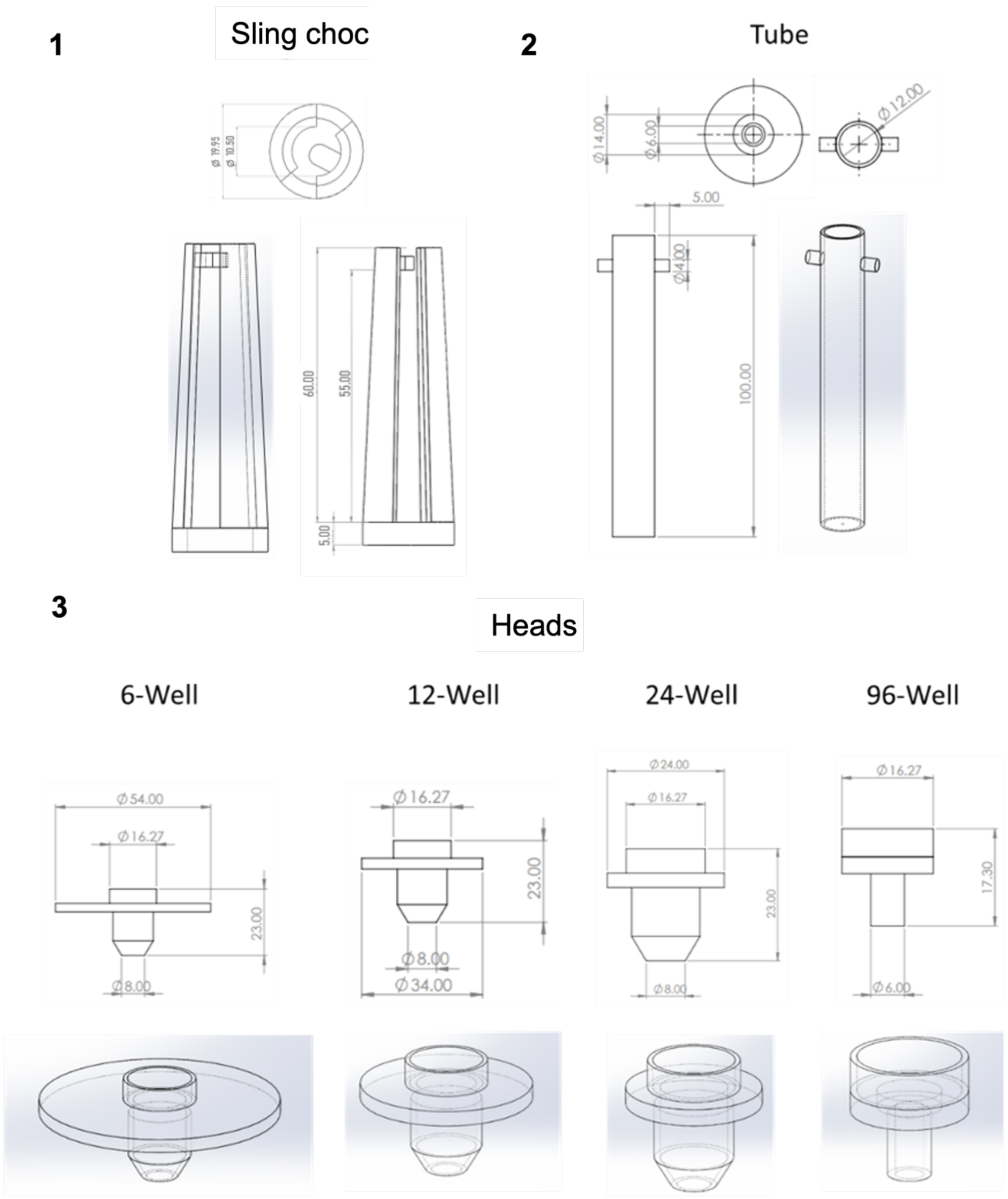

SI Fig. 1. Dimensions of 3D printed parts in mm. (1) Sling choc – this part ensure that every time the rubber will be starched exactly to the same distance. (2) tube TBI-ID (3) heads - modular design which fit any standard culture tool.
