# Supplementary material for: Traumatic Brain Injury in a Well: A Modular Three-Dimensional Printed Tool for Inducing Traumatic Brain Injury In vitro
Source: Neurotrauma Rep. 2023 Apr 20;4(1):255–66. doi: 10.1089/neur.2022.0072 (PMC10122253; doi:10.1089/neur.2022.0072)
Supplement: Supplemental data [file Suppl_TableS1.pdf]

**Height \ Weight**	**40 gr**	**50 gr**	**60 gr**
**1 cm**	3.922 mJ	4.903 mJ	5.883 mJ
**5 cm**	19.61 mJ	24.51 mJ	29.41 mJ
**10 cm**	39.22 mJ	49.03 mJ	58.83 mJ
**15 cm**	58.84 mJ	73.54 mJ	88.25 mJ

SI Table 1. Potential energy generated by different weights and heights distance.
