# Supplementary material for: Traumatic Brain Injury in a Well: A Modular Three-Dimensional Printed Tool for Inducing Traumatic Brain Injury In vitro
Source: Neurotrauma Rep. 2023 Apr 20;4(1):255–66. doi: 10.1089/neur.2022.0072 (PMC10122253; doi:10.1089/neur.2022.0072)
Supplement: Supplemental data [file Suppl_FigS3.pdf]

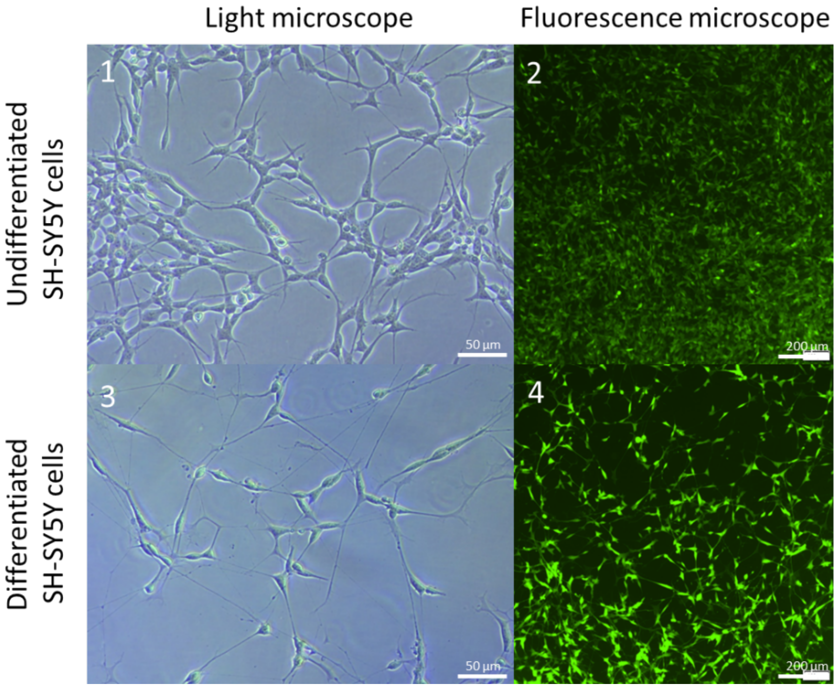

SI Fig. 3. SH-SY5Y cell differentiation. Representative images by light microscopy (1, 3) and fluorescence (GFP labeled) microscopy images (2, 4) of SH-SY5Y cells before (1, 2) and after (3, 4) differentiation to "neuron-like cells".
