# Supplementary material for: Traumatic Brain Injury in a Well: A Modular Three-Dimensional Printed Tool for Inducing Traumatic Brain Injury In vitro
Source: Neurotrauma Rep. 2023 Apr 20;4(1):255–66. doi: 10.1089/neur.2022.0072 (PMC10122253; doi:10.1089/neur.2022.0072)
Supplement: Supplemental data [file Suppl_TableS2.pdf]

model	study	result	Our model	Ref
**No cell**				
no cell	simulation work performed to study collapse of nanobubbles exposed to shock wave, sonoporation of biological membrane<br>(systems containing a bubble of D = 40 nm with up = 0.2, 0.3, 0.7 and 1.0 km s1)	model simulation BBB tight junctions after shock wave	*In vitro* model of BBB with<br>BMEC monolayer: TEER measurement and viability **(Figure 6)**	(Adhikari et al. 2016)
no cell	computational macroscale simulations predicting blast-induced intracranial fluid cavitation and microscale model	axon cores suffer significantly lower shear stresses from proximal bubble collapse than does their myelin sheathing. Simulations predict damage to myelin sheathing,<br>-> degrades axonal electrical transmissibility and general health of the white matter structures in the brain	Axonal morphology of SH-SY5Y cells after impact **(Figure 3)**<br><br>Primary neurons spontaneous electrical **(Figure 5)**	(Haniff and Taylor 2017)

no cell	physical head model with and without the brain agar phantom during impact.	found pressure vibration caused by the collapse of bubbles. It may be possible that the cavitation causes brain injury.		(Yusuke Kurosawa et al. 2009)
no cell	The damage was observed on a 9 μm thick aluminum foil attached to a glass substrate	the collapse of a bubble in the presence of shear flow, where most of the damage is created by the microjet mechanism. Shear flow influences the erosive potential of the bubble collapse.	High-speed images of bubbles formation **(Figure 2)**	(Dular et al. 2019)
**Cells**				
cells	3D Collagen Gel Preparation for Neuronal Compression	Morphological injury assessment after compressive impact. Analysis of the local and mean axial strains. (%) dead neurons. Cell permeability measurements	Axonal morphology of SH-SY5Y cells after impact and damaged area **(Figure 3, 4)**<br><br>Neurons viability, functionality **(Figure 5)**<br><br>BBB viability Teer measurement **(Figure 6)**	(Bar-Kochba et al. 2016)
Tissue	Exposure to impulse noise,	photographs of the shock/bubble interaction. Pressure and propagation of the blast. Histological specimen showing the damaged area after application of	High-speed images of bubbles formation **(Figure 2)**<br><br>SH-SY5Y cells damaged area after impact **(Figure 4)**	(Nakagawa et al. 2011)

		an underwater SW through the cranial window (10 MPa)		
Hippocampi were excised and sectioned into 400 µm thick slices + endothelial monoculture model of the BBB	blast injury: air shock wave into a fast-rising pressure	Characterization of the open shock tube: air/ fluid duration, air/ fluid impulse, pressure. (%) cell death tissue + BBB. Teer	Axonal morphology of SH-SY5Y cells after impact and damaged area **(Figure 3, 4)**<br><br>Neurons viability, functionality **(Figure 5)**<br><br>BBB viability Teer measurement **(Figure 6)**	(Effgen et al. 2012)
BBB: Brain endothelial monolayers	blast injury: air shock wave into a fast-rising pressure	Characterization: pressure, duration, impulse, peak overpressure, TEER, Immunostaining of ZO-1, cell detachment quantification	Axonal morphology of SH-SY5Y cells after impact and damaged area **(Figure 3, 4)**<br><br>Neurons viability, functionality **(Figure 5)**<br><br>BBB viability Teer measurement **(Figure 6)**	(Hue et al. 2013)
		**Whole organs**		
Adult Rat Brain	Exposure to impulse noise,198 dB (198 dB relative to 20 mPa) or 202 dB.	induces expression of gene c-Jun (play a role in the initiation of neuronal death, and apoptosis): TUNEL staining and immunohistochemical staining	Axonal morphology of SH-SY5Y cells after impact and damaged area **(Figure 3, 4)**<br><br>Neurons viability, functionality **(Figure 5)**	(Säljö et al. 2002)

rats (Lung injury study)	open-ended shock tube producing a short-duration low-amplitude shockwave:<br>11.5, 35 or 66 kPa static overpressure (nitrogen)	TUNEL-positive cells for the cortex,<br>hippocampus, corpus callosum, and periventricular region.<br>Western blot: heavy neurofilament, (NF200), and calpain-mediated spectrum<br>breakdown products (SBDP)<br>compound action potential (CAP) recordings	Neurons functionality recording **(Figure 5)**	(Park et al. 2011)

SI Table 2. Comparison of the known TBI models and pathologies to the TBI-ID .

Publication bibliography

Adhikari, Upendra; Goliaei, Ardeshir; Berkowitz, Max L. (2016): Nanobubbles, cavitation, shock waves and traumatic brain injury. In *Physical chemistry chemical physics : PCCP* 18 (48), pp. 32638–32652. DOI: 10.1039/c6cp06704b.

Bar-Kochba, Eyal; Scimone, Mark T.; Estrada, Jonathan B.; Franck, Christian (2016): Strain and rate-dependent neuronal injury in a 3D in vitro compression model of traumatic brain injury. In *Scientific reports* 6, p. 30550. DOI: 10.1038/srep30550.

Dular, Matevž; Požar, Tomaž; Zevnik, Jure; Petkovšek, Rok (2019): High speed observation of damage created by a collapse of a single cavitation bubble. In *Wear* 418-419, pp. 13–23. DOI: 10.1016/j.wear.2018.11.004.

Effgen, Gwen B.; Hue, Christopher D.; Vogel III, Edward; Panzer, Matthew B.; Meaney, David F.; Bass, Cameron R.; Morrison III, Barclay (2012): A multiscale approach to blast neurotrauma modeling: part II: methodology for inducing blast injury to in vitro models. In *Frontiers in neurology* (3). DOI: 10.3389/fneur.2012.00023.

Haniff, S.; Taylor, P. A. (2017): In silico investigation of blast-induced intracranial fluid cavitation as it potentially leads to traumatic brain injury. In *Shock Waves* 27 (6), pp. 929–945. DOI: 10.1007/s00193-017-0765-1.

Hue, Christopher D.; Cao, Siqi; Haider, Syed F.; Vo, Kiet V.; Effgen, Gwen B.; Vogel, Edward et al. (2013): Blood-brain barrier dysfunction after primary blast injury in vitro. In *Journal of neurotrauma* 30 (19), pp. 1652–1663. DOI: 10.1089/neu.2012.2773.

Nakagawa, Atsuhiro; Manley, Geoffrey T.; Gean, Alisa D.; Ohtani, Kiyonobu; Armonda, Rocco; Tsukamoto, Akira et al. (2011): Mechanisms of primary blast-induced traumatic brain injury: insights from shock-wave research. In *Journal of neurotrauma* 28 (6), pp. 1101–1119. DOI: 10.1089/neu.2010.1442.

Park, Eugene; Gottlieb, James J.; Cheung, Bob; Shek, Pang N.; Baker, Andrew J. (2011): A model of low-level primary blast brain trauma results in cytoskeletal proteolysis and chronic functional impairment in the absence of lung barotrauma. In *Journal of neurotrauma* 28 (3), pp. 343–357. DOI: 10.1089/neu.2009.1050.

Säljö, Annette; Bao, Feng; Jingshan, Shi; Hamberger, Andreas; Hansson, Hans-Arne; Haglid, Kenneth G. (2002): Exposure to Short-Lasting Impulse Noise Causes Neuronal c-Jun Expression and Induction of Apoptosis in the Adult Rat Brain. In *Journal of neurotrauma* 19 (8), pp. 985–991.

Yusuke Kurosawa; Kazuo Kato; Satoshi Saito (2009): 2009 annual international conference of the IEEE Engineering in Medicine and Biology Society. EMBC 2009 ; Minneapolis, Minnesota, USA, 3 - 6 September 2009. Piscataway, NJ: IEEE.
